# Delirium detection using wearable sensors and machine learning in patients with intracerebral hemorrhage

**DOI:** 10.3389/fneur.2023.1135472

**Published:** 2023-06-09

**Authors:** Abdullah Ahmed, Augusto Garcia-Agundez, Ivana Petrovic, Fatemeh Radaei, James Fife, John Zhou, Hunter Karas, Scott Moody, Jonathan Drake, Richard N. Jones, Carsten Eickhoff, Michael E. Reznik

**Affiliations:** ^1^Brown Center for Biomedical Informatics, Brown University, Providence, RI, United States; ^2^IMDEA Networks Institute, Madrid, Spain; ^3^Department of Neurology, Brown University, Providence, RI, United States; ^4^Department of Psychiatry, Brown University, Providence, RI, United States

**Keywords:** delirium, neurocritical care, stroke, intracerebral hemorrhage, actigraphy, machine learning, wearable electronic devices

## Abstract

**Objective:**

Delirium is associated with worse outcomes in patients with stroke and neurocritical illness, but delirium detection in these patients can be challenging with existing screening tools. To address this gap, we aimed to develop and evaluate machine learning models that detect episodes of post-stroke delirium based on data from wearable activity monitors in conjunction with stroke-related clinical features.

**Design:**

Prospective observational cohort study.

**Setting:**

Neurocritical Care and Stroke Units at an academic medical center.

**Patients:**

We recruited 39 patients with moderate-to-severe acute intracerebral hemorrhage (ICH) and hemiparesis over a 1-year period [mean (SD) age 71.3 (12.20), 54% male, median (IQR) initial NIH Stroke Scale 14.5 (6), median (IQR) ICH score 2 (1)].

**Measurements and main results:**

Each patient received daily assessments for delirium by an attending neurologist, while activity data were recorded throughout each patient's hospitalization using wrist-worn actigraph devices (on both paretic and non-paretic arms). We compared the predictive accuracy of Random Forest, SVM and XGBoost machine learning methods in classifying daily delirium status using clinical information alone and combined with actigraph data. Among our study cohort, 85% of patients (*n* = 33) had at least one delirium episode, while 71% of monitoring days (*n* = 209) were rated as days with delirium. Clinical information alone had a low accuracy in detecting delirium on a day-to-day basis [accuracy mean (SD) 62% (18%), F1 score mean (SD) 50% (17%)]. Prediction performance improved significantly (*p* < 0.001) with the addition of actigraph data [accuracy mean (SD) 74% (10%), F1 score 65% (10%)]. Among actigraphy features, night-time actigraph data were especially relevant for classification accuracy.

**Conclusions:**

We found that actigraphy in conjunction with machine learning models improves clinical detection of delirium in patients with stroke, thus paving the way to make actigraph-assisted predictions clinically actionable.

## 1. Introduction

Delirium occurs frequently in critically ill patients and has consistently been associated with higher mortality and worse overall outcomes (1). However, the diagnosis and detection of delirium remains challenging in many patient populations, as existing screening tools are unreliable in patients with stroke (2), neurocritical illness (3), and dementia (4). Further, because the diagnosis and detection of delirium relies on bedside testing, identifying at-risk patients across all hospital settings is highly labor intensive. Automated methods of delirium screening would therefore fill a critical need in the care of patients whose delirium may otherwise go undetected due to superimposed neurologic deficits, and potentially for critically ill patients as a whole.

We have previously described fluctuations of consciousness as a potential behavioral biomarker that corresponds to delirium in patients with stroke while also potentially identifying cases of delirium that went undetected by conventional screening tools (5). Additionally, we have found that these fluctuations may correspond to long-term outcomes in a subset of patients with hemorrhagic stroke (6). However, determining levels of consciousness still depends on frequent bedside assessments and may therefore be prohibitive. Given that motor activity is often heavily factored into clinical measurements of consciousness (7), and that psychomotor changes are a hallmark of delirium (8), continuous measurements of motor activity may represent a promising behavioral biomarker to aid in delirium detection.

Various devices for measuring motor activity exist, though wearable sensors such as wrist actigraphs are especially appealing due to their ease of use and relatively low cost. Such devices are commonly used in outpatient sleep medicine settings (9), and have increasingly been utilized in studies attempting to measure physical activity (10) and sleep (11) in the intensive care unit (ICU). However, actigraphy in ICU settings may be contaminated by externally mediated activity arising from clinical care. As a result, although wrist actigraphs have also been considered as a potential means of predicting delirium, existing studies have thus far shown mixed results using conventional statistical methods (12–14).

In this study, we aimed to mitigate some of this externally mediated artifact by collecting actigraphy data from a cohort of critically ill patients with hemiparesis due to acute intracerebral hemorrhage (ICH). Owing to marked differences in activity profiles between paretic and non-paretic sides, we aimed to leverage actigraphy data from both wrists as a within-patient control. Further, the heterogeneous nature of individual activity profiles and delirium cases lends itself to advanced analysis using machine learning-based techniques. We therefore designed this study to test the feasibility of this novel approach.

## 2. Materials and methods

### 2.1. Study population

We prospectively screened all patients admitted to Rhode Island Hospital's Neurocritical Care Unit (NCCU) or Stroke Unit (SU) with acute intracerebral hemorrhage (ICH) over a 1-year period from 2018 to 2019 for potential enrollment. We included patients with moderate-to-severe supratentorial ICH [National Institutes of Health (NIH) stroke scale ≥ 5] and hemiparesis, specifically focusing on supratentorial ICH because of the higher likelihood of cognitive symptoms occurring in conjunction with motor symptoms as compared to patients with brainstem or cerebellar hematomas. We excluded patients with previous limb amputation or significant pre-morbid functional disability requiring assistance with their daily activities (as assessed using the modified Rankin Scale), as well as patients with devastating strokes considered to have a high likelihood of mortality. All patients were enrolled within 72 h of admission.

### 2.2. Delirium assessments

Daily delirium assessments were performed each afternoon by an attending neurocritical care or behavioral neurologist, with the exception of weekends and holidays. Delirium was diagnosed according to Diagnostic and Statistical Manual of Mental Disorders, Fifth Edition (DSM-5) criteria (15): disturbances in attention and awareness (often accompanied by disturbances in other cognitive domains, such as psychomotor slowing or agitation, disorientation, disorganized thinking, impaired executive function, or perceptual disturbance) that develop over a short period of time and tend to fluctuate, represent a change in function, and are due to an underlying medical condition or toxic/withdrawal syndrome. Assessments were supplemented by interviews and history obtained from patients' nurses and clinical providers, family members, and the medical chart. Joint adjudication sessions were held between the participating neurologists to obtain consensus on delirium diagnoses for each patient.

### 2.3. Actigraph data collection

Wrist actigraphs (Micro Motionlogger, Ambulatory Monitoring, Inc., Ardsley, NY) were placed on both wrists for each patient and left in place for the duration of their stay in the NCCU or SU. Actigraphs were otherwise only purposefully removed in anticipation of magnetic resonance imaging scans as a safety precaution and were then replaced thereafter.

Each actigraph was configured to collect activity data in 1-min epochs. These data were then aggregated by proprietary algorithms from the Action4 software package (Ambulatory Monitoring, Inc.) into two distinct measurements: Zero Crossing Mode (ZCM), which measures the frequency of movement by counting the number of times per epoch that the signal crosses a threshold set near zero; and Proportional Integration Mode (PIM), which calculates the area under the curve for the acceleration signal during each epoch, and therefore discriminates between different intensities of motion. Data were subsequently downloaded using the Action W-2 software package (Ambulatory Monitoring, Inc.). A sample of a patient's actigraph data is provided in Supplementary material 1.

### 2.4. Additional clinical data

All data related to standard clinical stroke care were prospectively collected in a REDCap database (Vanderbilt University, Nashville, TN) (16, 17). These data included patient demographics, comorbidities, admission NIH Stroke Scale (NIHSS) score, neuroimaging, and other diagnostic testing. ICH-related clinical predictors, including hematoma location, size, and ICH score were adjudicated by two attending neurologists with board certification in neurocritical care and/or vascular neurology until consensus was achieved.

### 2.5. Data analysis

The task of recognizing episodes of delirium was delineated as a supervised machine learning problem: Given a set of features *X* derived from patient data, predict delirium status as the dependent outcome variable *Y*. Because delirium status was measured on a 24-h basis, patient data were preprocessed such that individual features were aggregated into a day-level unit of analysis.

### 2.6. Actigraph data pre-processing

Raw data for each patient consisted of two parts: raw actigraph measurements, which had minute-to-minute variability, and clinical data, which remained relatively static over the course of each patient's hospitalization (in the case of demographic and stroke-specific data) or over the course of a day (in the case of mechanical ventilation status). Actigraph data for a given 24-h block were partitioned into four groups: full-day (1 to 1 P.M.), morning (6 A.M. to 1 P.M.), afternoon/evening (1 to 10 P.M.), and night-time (10 P.M. to 6 A.M.) epochs. Pre-processing also included normalization of actigraph data prior to classification.

### 2.7. Actigraph feature extraction

**Figure 1:**
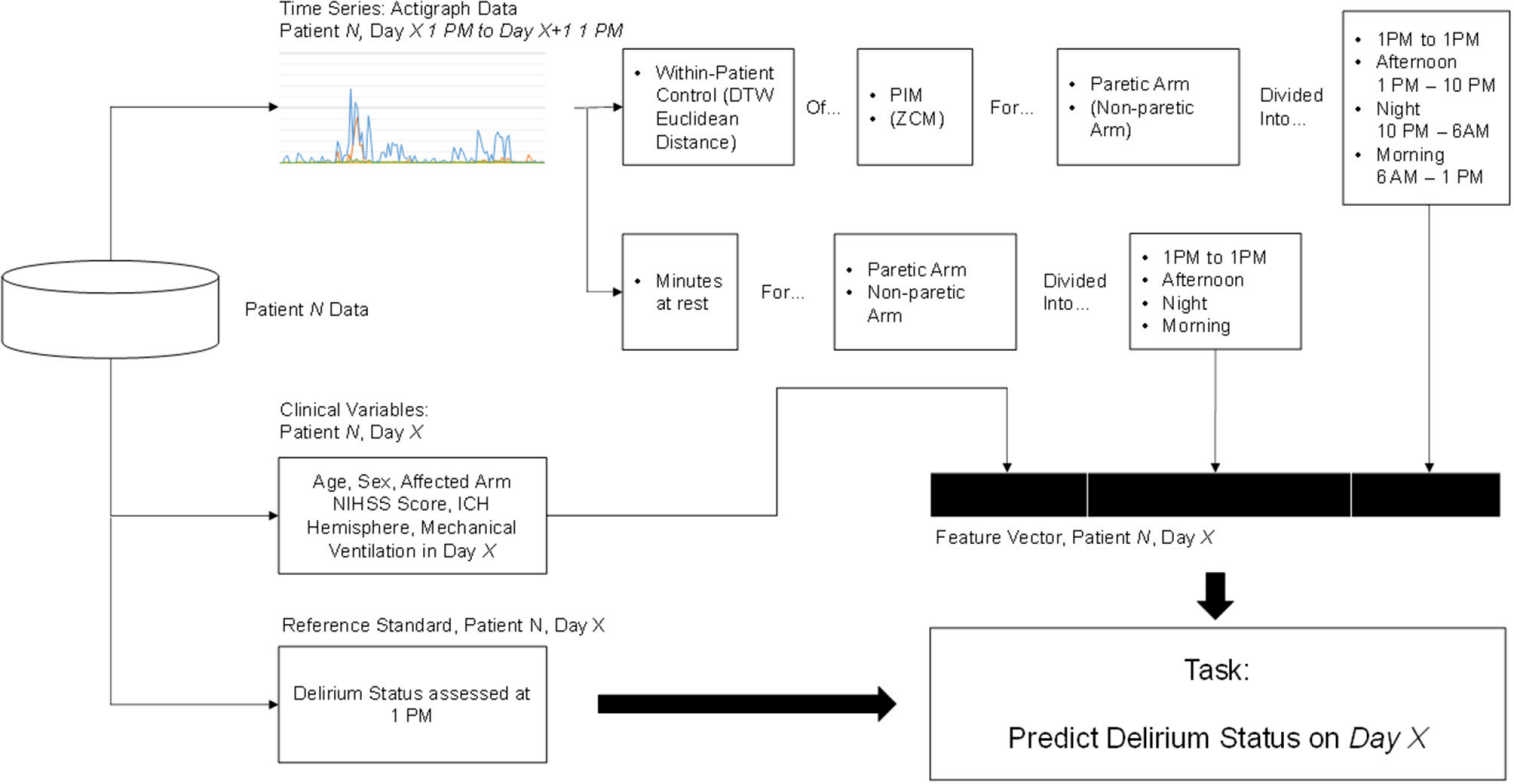
Feature extraction and classification process. Elements in parentheses were initially explored but ultimately discarded because they did not contribute to higher prediction accuracy.

We then aimed to extract clinically meaningful information from the actigraph data to include as features in our delirium prediction models. Figure 1 summarizes the feature extraction process, which culminated in our calculation of two key features from the actigraph data: minutes at rest and within-patient dynamic time warping (DTW).

We defined minutes at rest as the daily proportion of PIM measurements equal to zero in both paretic and non-paretic arms. This feature was chosen due to the importance of psychomotor slowing and inactivity in the diagnosis of delirium (18). It may also provide a helpful estimate of sleep-wake disturbance, a common symptom of delirium (18), though actigraphy likely overestimates actual sleep time in hospital and ICU settings (10).

**Figure 2:**
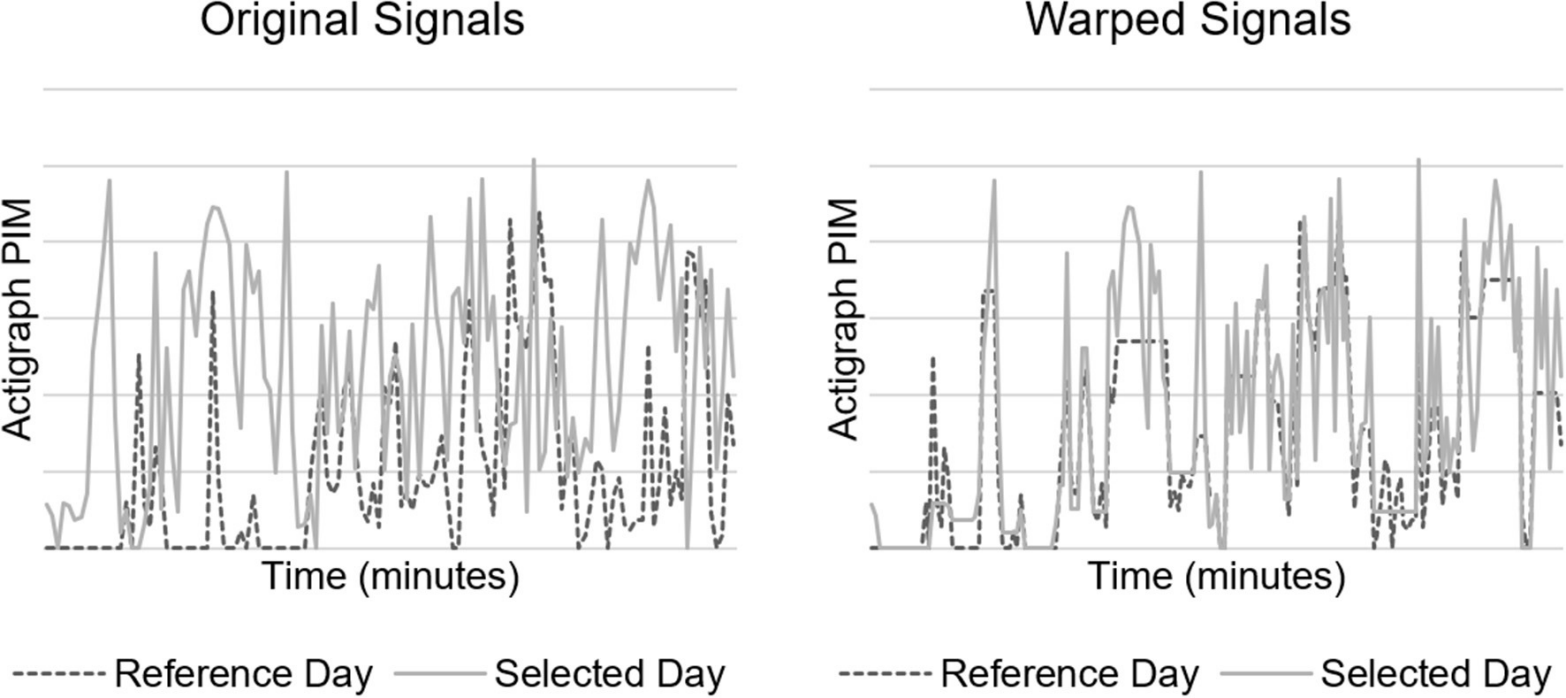
Dynamic time warping (DTW) example. Original **(left)** and minimal Euclidean distance warped **(right)** signals of 2 h of actigraph data.

We also implemented a within-patient control feature calculated as the minimal Euclidean distance of actigraph data with DTW (19). DTW offers a measure of similarity between two temporal sequences that vary in speed. We hypothesized that warping the actigraph signal would facilitate direct comparison of movements caused by routines occurring at regular intervals (e.g., nursing assessments and nursing care), but not necessarily at identical intervals each time, which would address intra-patient variability in our dataset. A higher Euclidean distance suggests a larger difference between the signal in question and the reference. Figure 2 depicts a DTW example.

We considered two separate within-patient control references: using the non-paretic arm as reference, and using the paretic arm in the first day labeled as non-delirious as reference. For consistently delirious patients without non-delirious days, we used the first day of data instead. Our rationale for using within-patient control references is that a patient would serve as their own best baseline to measure changes in movement, and that assessing movement in both arms could function as a surrogate for whole body movement. For instance, increased movement in both paretic and non-paretic arms would suggest externally mediated whole-body movement (e.g., for nursing care), while increased movement in only the non-paretic arm would be more suggestive of patient-initiated limb movement.

### 2.8. Clinical features

Finally, we included relevant demographics and stroke-specific clinical features in our delirium prediction models. These variables included age, sex, NIHSS scores, and ICH features including hematoma volume, location, and presence of intraventricular hemorrhage, many of which have been described as risk factors for post-stroke delirium in prior studies (20, 21). In addition, we included day-to-day mechanical ventilation status as a dynamic variable in our models, as this was presumed to affect the amount of patient movement (e.g., from sedation).

### 2.9. Train/test split

To account for limited data set size and high variance among enrolled patients' measurements, we used 500 bootstrapping iterations. During each iteration, data were split into non-overlapping training (80% of patients) and test sets (20% of patients), ensuring that no individual patient's observations were included in both the training and test set. Model training and evaluation were separately performed for each of these 500 random train/test splits. Means and standard deviations across the bootstrapping iterations were reported for metrics including accuracy, balanced accuracy, Receive Operating Characteristic Area Under the Curve (ROC-AUC), and F1-score.

### 2.10. Machine learning models

A number of alternative machine learning models were trained based on the previously described feature vectors. These included Random Forests, Support Vector Machines (SVM), and Extreme Gradient Boosting (XGBoost). Several filtering techniques were also explore to address the potential impact of patient intervariability, including bandpass and Savitzky-Golay filtering. However, we found no filtering method that improved performance and thus proceeded without any further data preprocessing. We provide the best results obtained by these algorithms, achieved using XGBoost with the following hyperparameters, all of which were tuned via cross-validation on the training set: learning rate 0.03, maximum tree depth 5, minimum child weight 1, subsample fraction 0.8, and column fraction 0.8. Models were trained to detect whether a patient had been delirious at any time during a 24-h period using different subsets of data sources: (1) clinical data only, (2) clinical and actigraph data using the non-paretic arm as reference, and (3) clinical and actigraph data using the first non-delirious day of the paretic arm as reference. We trained our models on a computer with an Intel 8700K CPU and 64 GB of RAM. Using this system, data preparation took ~10 min, and model training and testing took 1–2 min.

## 3. Results

### 3.1. Patient characteristics

A total of 40 patients who met eligibility criteria were recruited for this study. A patient flow diagram is provided in Supplementary material 1. To ensure model consistency, we discarded partial actigraph data from the day of enrollment, which also removed the substantial artifact associated with actigraph setup. This led to the exclusion of one patient who was enrolled and discharged prior to recording a full day of actigraph data. As a result, our final cohort comprised 39 patients with a total of 296 days of actigraph monitoring (see Table 1 for baseline characteristics). Among this cohort, 85% of patients (*n* = 33) had delirium at some point during their hospitalization, including 15 patients who had delirium for the entire duration of monitoring, while 15% of patients (*n* = 6) never had delirium; 71% (*n* = 209) of all monitoring days represented days with delirium.

**Table 1:** Baseline characteristics and delirium features for patients with intracerebral hemorrhage (ICH) enrolled in this study.

**Demographics**	
Age, years, mean (SD)	71.4 (12.2)
Male, *n* (%)	21 (53.9%)
White, *n* (%)	38 (97.5%)
**ICH characteristics**	
Admission NIHSS score, median (IQR)	14.5 (6)
ICH score, median (IQR)	2 (1)
ICH volume, cc, mean (SD)	38.4 (24.1)
Intraventricular hemorrhage, *n* (%)	21 (53.9%)
**Location**, ***n*** **(%)**^a^	
Lobar	26 (66.7%)
Deep	14 (38.5%)
Mechanically ventilated, *n* (%)	8 (20.5%)
Ever delirious, *n* (%)	33 (84.6%)
Always delirious, *n* (%)	15 (38.5%)
Number of data collection days, median (IQR)	5 (9)
Delirium days, median (IQR)	3 (6)

^a^One patient presented with both lobar and deep ICH.

### 3.2. Delirium detection

Clinical data alone had low accuracy in detecting delirium on a day-to-day basis (Table 2). However, the addition of actigraph data yielded a significant improvement in accuracy (*p* < 0.001), with the highest recognition performance reaching an accuracy score of 74%. Using the first non-delirious day as DTW reference resulted in better accuracy than using the non-paretic arm as reference.

**Table 2:** Model performance for same-day delirium detection.

**Data**	**Balanced accuracy**	**Accuracy**	**F1**	**ROC AUC**
Clinical only	0.56 ± 0.14	0.62 ± 0.18	0.5 ± 0.17	0.56 ± 0.14
Clinical + minutes at rest	0.61 ± 0.1	0.69 ± 0.1	0.58 ± 0.11	0.61 ± 0.1
Clinical + non-paretic arm DTW	0.57 ± 0.1	0.63 ± 0.11	0.53 ± 0.11	0.57 ± 0.1
Clinical + non-paretic arm DTW + minutes at rest	0.63 ± 0.1	0.71 ± 0.1	0.61 ± 0.1	0.63 ± 0.1
Clinical + reference day DTW	0.66 ± 0.12	0.68 ± 0.12	0.61 ± 0.12	0.66 ± 0.12
Clinical + reference day DTW + Minutes at rest	0.65 ± 0.11	0.71 ± 0.11	0.62 ± 0.11	0.65 ± 0.11
Clinical + non-paretic arm DTW+ reference day DTW + minutes at rest	**0.68** **±0.11**	**0.74** **±0.1**	**0.65** **±0.1**	**0.68** **±0.11**

Best performing method bolded.

In a *post-hoc* analysis, we attempted to further filter activity that was presumed to be externally mediated (e.g., from nursing care) by removing certain actigraph data outliers. We defined these outliers as PIM values from the paretic arm that were 10 or more standard deviations higher than the median paretic arm PIM value. However, this procedure did not improve accuracy (accuracy 0.74, balanced accuracy 0.68, F1 0.65, ROC AUC 0.68).

**Figure 3:**
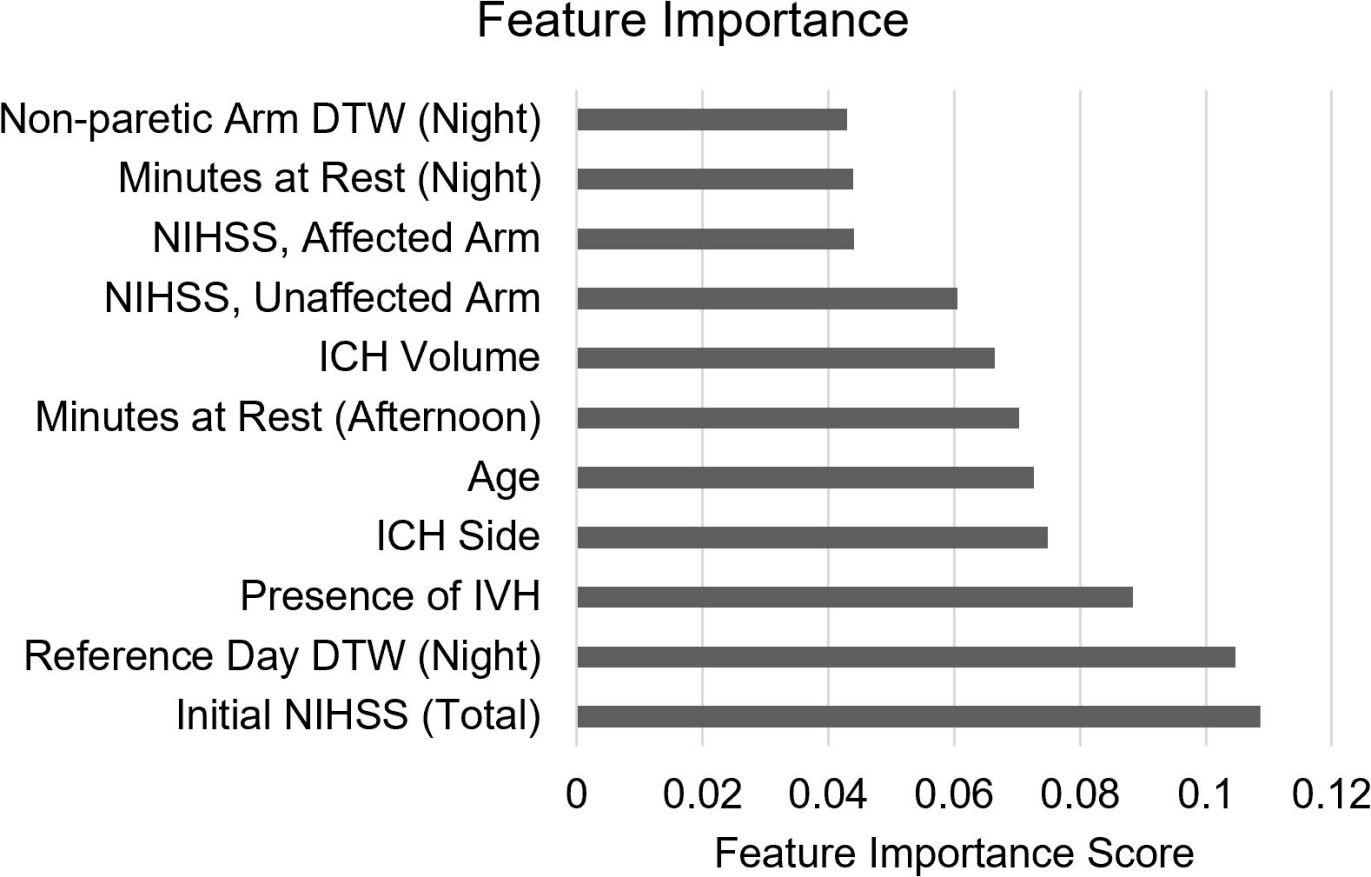
Individual feature importance for the final model according to XGBoost.

We were also interested in determining which individual features most contributed to model accuracy. This was done by analyzing each feature and the associated information gain it produced, averaged across each run of cross validation. This analysis suggests that night-time DTW actigraph data was an especially important feature, with minutes at rest during afternoon/evening and night-time periods also meaningfully contributing to prediction power (Figure 3).

## 4. Discussion

We found that detecting delirium using actigraphy and machine learning-based analysis was feasible and provided valuable information that significantly improved upon the accuracy of clinical data alone. Given the increasingly recognized impact of delirium on patients with stroke, with consequences ranging from withdrawal of life-sustaining treatment to decreased rates of rehabilitation utilization (22), the early and accurate recognition of delirium in these patients is paramount. Because existing delirium screening tools are unreliable in the setting of severe neurologic deficits, novel tools are needed in the clinical setting, and unconventional methods such as actigraph monitoring may be a promising way to address this gap (21).

The use of machine learning with actigraphy data has been previously implemented for sleep-wake detection with encouraging results (23–26), and methods using XGBoost have shown superior performance in classifying actigraph data (24). However, to the best of our knowledge, this is the first study that uses machine learning in combination with actigraphy data to assess delirium status in any patient population.

Theoretically, our study may also have relevance for non-neurologic patients (12, 27), though additional considerations would be required for patients with greater mobility. Although validated delirium screening tools exist in general critical care and hospitalized populations, they can be resource intensive and demand that nurses and other providers be appropriately trained in their use. Automated methods of delirium monitoring could therefore help offset the burden of a strained healthcare staff, especially methods that help monitor for fluctuations in activity and arousal that are characteristic of delirium. Additionally, adjunctive methods of delirium monitoring may also improve overall detection rates in other challenging patient populations, such as those with dementia.

Monitoring patients at night may be especially important in detecting delirium, a concept that is underscored by the significant contribution of night-time actigraph data to the accuracy of our delirium prediction models. Although the high incidence of nocturnal symptoms and sleep-wake disturbances associated with delirium is well known, these symptoms may often go undetected until they reach a point where they become obvious (i.e., severe agitation). It is possible that nocturnal symptoms could be detected sooner via frequent clinical assessments, which are the basis of neurological monitoring in neurocritically ill patients. However, there is increasing controversy regarding the optimal frequency of neuro checks, and it has been hypothesized that overly frequent neurological exams at night may contribute to delirium by leading to sleep fragmentation and overstimulation (28). On the other hand, the relatively unobtrusive nature of wearable sensors may provide valuable complementary information that could help detect delirium during especially high-risk time periods.

Our study is notable for its innovative techniques, including the use of machine-learning to analyze actigraph data and the use of within-patient controls via actigraphs worn on both paretic and non-paretic limbs. However, the study does have several limitations. First, actigraph data are limited by noise and artifacts caused by external movements such as nurse or provider-initiated movements (e.g., during repositioning or clinical examination). Although we excluded artifactual data associated with actigraph initiation from the day of admission and incorporated measurements from both paretic and non-paretic limbs to mitigate potential confounding, we could not definitively filter these externally-mediated movements further. However, their influence may have been relatively modest, as a *post-hoc* analysis using an outlier filtering method did not result in a meaningful difference in accuracy. Second, because we assessed delirium status only once per day, we may have missed shorter periods of delirium or non-delirium that would have allowed for closer correlation with actigraph data. Finally, our sample size was relatively small and had a class imbalance in favor of delirium-positive days, as many patients were rated as either always (or almost always) delirious or never delirious. Because deep learning requires that voluminous amounts of data be available for optimal results, future studies are needed to analyze data from larger cohorts of patients and further evaluate machine learning-based methods for detecting and predicting delirium. The methods described in this manuscript, paired with a larger dataset, may provide a resulting model that generalizes better and shows less variability in its output metrics.

## 5. Conclusions

We found that actigraphy in conjunction with machine learning models improves clinical detection of delirium in patients with stroke, thus paving the way to make actigraph-assisted predictions clinically actionable.

## Data availability statement

The raw data supporting the conclusions of this article will be made available by the authors, upon reasonable request.

## Ethics statement

The studies involving human participants were reviewed and approved by Lifespan Institutional Review Board 2 (ID #1126240). The patients/participants provided their written informed consent to participate in this study.

## Author contributions

MR, CE, and RJ: conceptualization and methodology. MR and CE: funding acquisition and supervision. AA, AGA, IP, FR, JF, JZ, HK, SM, JD, CE, and MR: investigation and analysis of data. AA, AGA, CE, and MR: drafting and/or revising significant portions of the manuscript. All authors contributed to the article and approved the submitted version.
